# Time-resolved grazing-incidence pair distribution functions during deposition by radio-frequency magnetron sputtering

**DOI:** 10.1107/S2052252519001192

**Published:** 2019-02-23

**Authors:** Martin Roelsgaard, Ann-Christin Dippel, Kasper Andersen Borup, Ida Gjerlevsen Nielsen, Nils Lau Nyborg Broge, Jan Torben Röh, Olof Gutowski, Bo Brummerstedt Iversen

**Affiliations:** a Center for Materials Crystallography, Department of Chemistry, Aarhus University, Aarhus, Denmark; b PETRA III, Deutsches Elektronen-Synchrotron, DESY, Hamburg, Germany

**Keywords:** pair distribution function, thin films, grazing-incidence PDF, magnetron sputtering

## Abstract

Characterization of local order in thin films is challenging with pair distribution function (PDF) analysis because of the minute mass of the scattering material. Here, it is demonstrated that reliable high-energy grazing-incidence total X-ray scattering data can be obtained *in situ* during thin-film deposition by radio-frequency magnetron sputtering. A benchmark system of Pt was investigated in a novel sputtering chamber mounted on beamline P07-EH2 at the PETRA III synchrotron. Robust and high-quality PDFs can be obtained from films as thin as 3 nm and atomistic modelling of the PDFs with a time resolution of 0.5 s is possible. In this way, it was found that a polycrystalline Pt thin film deposits with random orientation at 8 W and 2 × 10^−2^ mbar at room temperature. From the PDF it was found that the coherent-scattering domains grow with time. While the first layers are formed with a small tensile strain this relaxes towards the bulk value with increasing film thickness.

## Abstract 2

During thin-film deposition by radio-frequency magnetron sputtering reliable high-energy grazing-incidence X-ray scattering data are obtained. *In situ* pair distribution function analysis is achieved allowing the deposition process to be investigated.

## 1. Introduction   

Magnetron sputtering is a widely used physical vapour deposition technique to fabricate thin films, from a few nanometres to several micrometres in thickness and in large areas relevant for industrial applications. The most versatile form of sputtering is radio-frequency (RF) magnetron sputtering. Sputter deposition can fabricate films with structural ordering at different length scales: epitaxial, polycrystalline with and without texture, nanocrystalline or amorphous. While the structural analysis of crystalline and epitaxial thin films can make use of Bragg scattering, the nanocrystalline and amorphous films are more challenging to characterize with X-ray scattering as they do not show any sharp Bragg reflections. In addition, the undesired contributions from the substrate typically dominate the signal coming from the disordered films. Hence, the formation and growth processes are difficult to study in particular in the case of amorphous and nanocrystalline films. Many specialized sputtering chambers have been developed for *in situ* investigations of film growth at synchrotron sources using both small-angle X-ray scattering (SAXS) and X-ray diffraction (Schroeder *et al.*, 2015 ▸; Walter *et al.*, 2015 ▸; Payne *et al.*, 1992 ▸; Matz *et al.*, 2001 ▸; Williams *et al.*, 1992 ▸; Lee *et al.*, 2000 ▸; Couet *et al.*, 2008 ▸; Renaud *et al.*, 2009 ▸; Folkman *et al.*, 2013 ▸; Kaufholz *et al.*, 2015 ▸; Nicklin *et al.*, 2017 ▸). So far, to our knowledge, none of the reported *in situ* film-growth studies have addressed the local atomic structure.

Total scattering and pair distribution function (PDF) analyses have become popular tools to investigate local- to medium-range order in amorphous nanocrystalline or defect materials (Bøjesen & Iversen, 2016 ▸; Mancini & Malavasi, 2015 ▸; Young & Goodwin, 2011 ▸; Billinge & Levin, 2007 ▸). However, in the case of thin films, PDF analysis is still in its infancy because of the weak signal obtained from samples with intrinsically low film-to-substrate ratio. In fact, thin-film PDFs with a quality suitable for structural refinement were only reported in 2015 using transmission-mode measurements (Jensen *et al.*, 2015 ▸), and several additional papers on this technique have appeared since then (Nakamura *et al.*, 2017 ▸; Wood *et al.*, 2017 ▸; Shi *et al.*, 2017 ▸; Shyam *et al.*, 2016 ▸; Stone *et al.*, 2016 ▸). Quantitative PDF analysis is therefore possible from thin films in transmission geometry, but common to all of these studies are film thicknesses typically of hundreds of nanometres. On the other hand, grazing-incidence X-ray diffraction (GIXRD) provides an increased surface sensitivity and scattering intensity. Using grazing incidence in PDF analysis is a recent development, and is discussed in further detail elsewhere (Dippel *et al.*, 2019 ▸). Local- and medium-range order in Ta_2_O_5_ and VO_2_ were measured and modelled for films as thin as 50 nm using GIXRD (Stone *et al.*, 2016 ▸; Shyam *et al.*, 2016 ▸).

Here, we demonstrate time-resolved grazing-incidence PDF in a dedicated film-deposition chamber mounted at the P07-EH2 beamline at PETRA III, Hamburg, Germany, by studying RF magnetron sputtering of Pt, which is widely used *e.g.* as an electrode in functional films. In our study, it provides a strongly scattering, polycrystalline sample to analyse the PDFs during the first stages of film deposition.

## 2. RF magnetron sputtering unit for *in situ* PDF studies   

**Figure 1:**
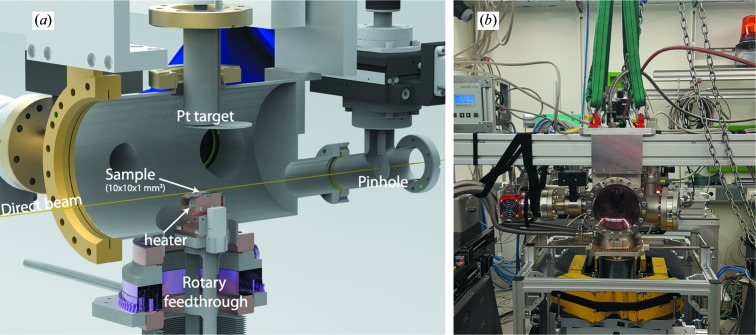
(*a*) A sketch of the sputter unit. The rotary feedthrough and bellows combination allows for free movement of the sample relative to the X-ray beam. Fast access to the sample is provided by an entry door with viewport from the right-hand side from the given perspective. A 1-inch sputter gun was fitted from the top with 75 mm target–substrate distance as adjusted by a spacer flange. Downstream of the glass entrance window the beam passes through a motorized pinhole in vacuum, before reaching the sample substrate. Further models and pictures of the setup from different angles are available in the Supporting information. (*b*) Photograph of the sputtering system mounted onto the surface diffractometer at P07-EH2 at PETRA III, DESY, Hamburg, Germany.

Previously a versatile and lightweight sputtering chamber for *in situ* studies of thin-film growth by powder XRD and GISAXS was reported (Walter *et al.*, 2015 ▸). To obtain the *Q* range required for PDF analysis, preferably of more than 20 Å^−1^, a large exit window with diameter 150 mm positioned 12 cm downstream from the sample position was used. Both 125 µm Kapton and PEEK windows are viable window materials to minimize background scattering, with the stiffer Kapton being preferable because of the smaller curvature of the window under vacuum. The window is sealed by a Viton-gasket, readily maintaining a baseline pressure of <10^−7^ mbar. The chamber design is depicted in Fig. 1 ▸. A motorized beamstop is positioned as close as possible to the window outside the pumped chamber. Background scattering from upstream components and the X-ray entrance window is removed by an in-vacuum pinhole with two translations between the entrance window and the sample. The combination of the in-vacuum pinhole and beamstop catching the Kapton-scattering leaves virtually no stray scattering from the direct beam.

In order to align the sample in grazing incidence, three translations and three rotations are required. Considering the high photon energy of 100 keV (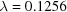 Å) and the resulting small scattering angles, the alignment has to be carried out at high precision. The sample was moved by the surface diffractometer at the P07-EH2 end station, which fulfils these requirements. For this purpose, the sample mount and vacuum chamber are connected through a rotary feedthrough and bellows combination that enables movement of the sample, while the rest of the chamber stays fixed. The setup was designed in such a way that the diffractometer can handle the load from the vacuum equipment and torque from the rotation.

To monitor and control the temperature at the sample position a thermocouple was mounted in the steel-based sample holders. A heater consisting of a 250 W halogen bulb was used that reliably sustained temperatures up to 1023 K at the sample position. Excess heat was removed by water cooling connected to the copper block that the sample holder is clamped onto. The sample holder is placed in an elevated tower mounted on a base-plate with feedthroughs for heating, water cooling and thermocouple connections.

The sputter gun is a 1-inch gun (type ST 10) from AJA International Inc. with a 100 W RF power supply. The gas inlet is controlled by two mass-flow controllers from MKS, allowing a gas-inlet flow of up to 10 sccm (standard cm^3^ min^−1^) O_2_ and 20 sccm Ar. The pressure is stabilized by an active gauge valve from MKS. The system is fully remote controlled by home-built software based on *PyTango* and *PyQt4*.

## 3. Experimental   

In a typical experiment the chamber was pumped to a baseline pressure of 5 × 10^−5^ mbar prior to sputter deposition. Pt films were deposited from a 1-inch 99.99% polycrystalline Pt target, onto 10 × 10 × 1 mm^3^ Heraeus HOQ310 glass (fused quartz) substrates heated to either 300 or 473 K isothermally. The sample surface was aligned parallel to the X-ray beam and subsequently the incidence angle fixed at α = 0.040° on the surface diffractometer. RF sputtering was performed with a forward power of 8 W in Ar at 0.02 or 0.05 mbar. Deposition continued until the films reached a thickness of up to ∼30 nm.

A PerkinElmer XRD1621 detector was used to collect two-dimensional diffraction patterns by the rapid-acquisition PDF technique (Chupas *et al.*, 2003 ▸). A fixed sample-to-detector distance of 450 mm was chosen, which is restricted by the bulky setup, resulting in a *Q* range up to 22 Å^−1^ with an X-ray energy of 98.5 keV. The beam was focused to 500 × 3 µm^2^ horizontal and vertical (Bertram *et al.*, 2016 ▸), respectively, at the sample position by Si compound refractive lenses. The 2D images were reduced by azimuthal integration to 1D 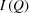 in *PyFAI* (Ashiotis *et al.*, 2015 ▸), and Fourier analysis was performed in *PDFgetX3* on PDFs (Juhás *et al.*, 2013 ▸).

X-ray reflectivity (XRR) data were collected either on the beamline after deposition or with a laboratory Rigaku SmartLab system equipped with a Co *K*α source with a parallel-beam (2 × 0.5 mm) option. The X-ray reflectivity was refined using *LSFit* by O. Seeck (DESY, Hamburg).

## 4. Results and discussion   

### 4.1. Thin-film deposition   

**Figure 2:**
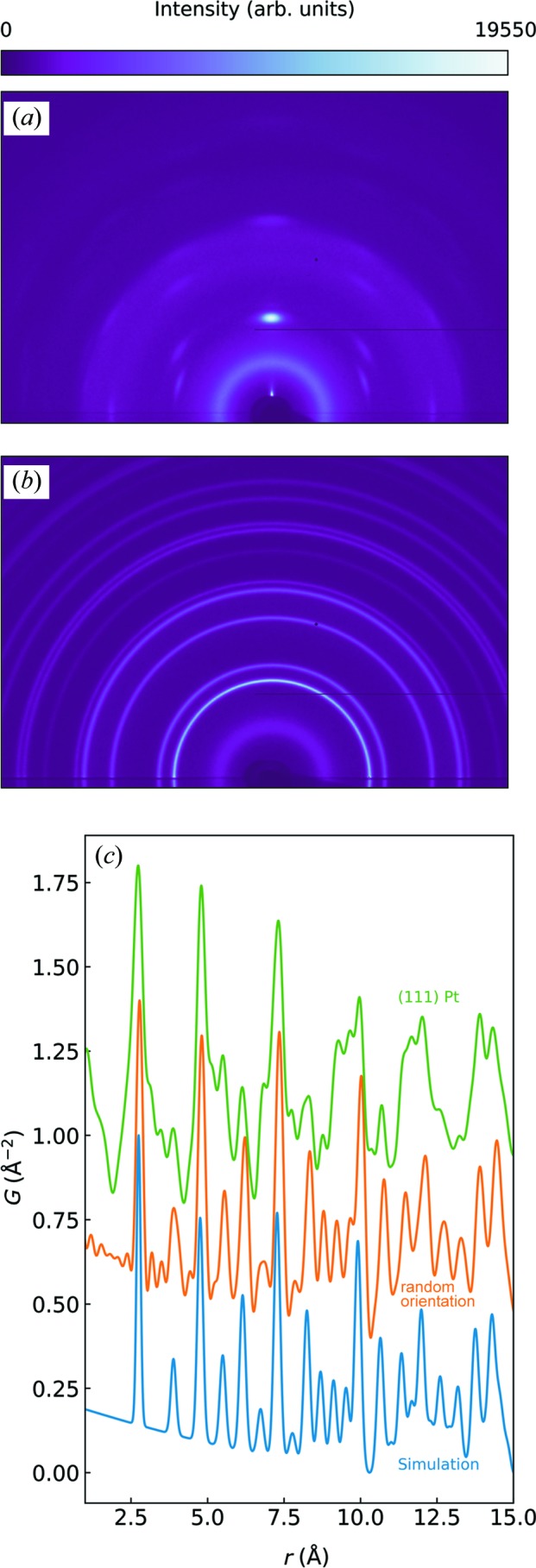
(*a*) and (*b*) show 2D scattering for two Pt samples with and without (111) fibre texture, respectively. The scattering intensity below the substrate horizon is shadowed by the sample holder and the substrate itself. The corresponding PDFs are shown in (*c*) and compared with simulated data of f.c.c. Pt (blue).

First we demonstrate how the microstructure and crystallographic orientation of the Pt film depend on the sputter process as well as the substrate temperature. On amorphous substrates the closest packed plane in the crystal structure, such as the (111) plane in f.c.c.-structured Pt, tends to orient normal to the substrate surface (Kawamura *et al.*, 2004 ▸; Rodriguez-Navarro, 2001 ▸). Other orientations such as (100) Pt have been achieved *e.g.* by introducing oxygen into the sputter gas (Kim *et al.*, 1999 ▸; McBride *et al.*, 1991 ▸); however, such films are typically of low crystallinity or have oxygen incorporated. In the present work, the sputter parameters were chosen such that the deposition rate was ∼1.1 Å s^−1^ as observed by XRR (measured *ex situ*). By lowering the substrate temperature and the kinetic energy of the incoming ions by increasing the pressure, a randomly oriented film can also be obtained. The two extreme cases are demonstrated with the 2D diffraction images in Fig. 2 ▸. For the data shown in Fig. 2 ▸(*a*), the sample was prepared at a pressure of 0.020 mbar and a temperature of 473 K, whereas for the data shown in Fig. 2 ▸(*b*), the sample was prepared at a pressure of 0.050 mbar and 300 K. Texture is typically characterized by pole figures, which visualize the orientation distribution of a given plane (see the Supporting information for pole figures of the textured sample). In the 2D diffraction patterns, the strong (111) reflection clearly shows that the crystallites have a preferred orientation in the vertical direction. In PDF analysis the 2D diffraction patterns are usually reduced to 1D by azimuthal integration and hence this information is lost. In the present work, only the scattered intensity above the sample horizon was used. The PDFs of the randomly oriented as well as the (111) Pt films are shown in Fig. 2 ▸(*c*). Even though the peak positions are retained in the textured PDF, the relative peak intensities are off. There are also some apparent negative dips in the PDF resulting in an overestimation of the 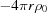 slope at low *r* as compared with the simulated PDF of Pt (blue). These effects lead to artefacts in the PDF, 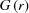, that cannot be accounted for without introducing orientation *e.g.* in the form of the orientation distribution function in 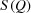, as recently discussed by Gong & Billinge (2018 ▸). However, it is outside the scope of the present work to treat texture in PDF in further detail.

### 4.2. Obtaining the PDFs   

**Figure 3:**
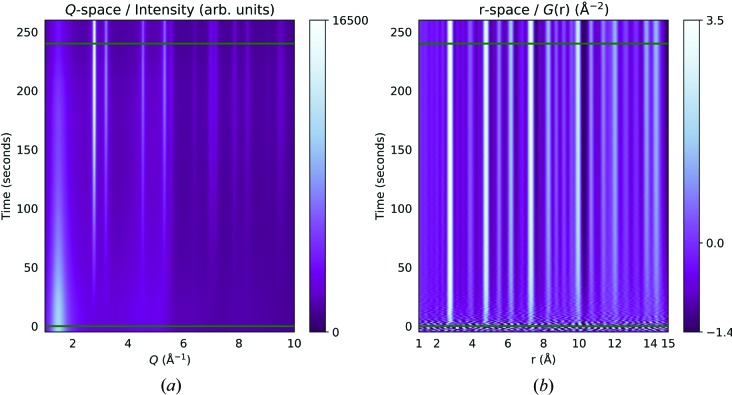
GIXRD and GIPDF as functions of time in (*a*) *Q* space and (*b*) real (*r*) space, respectively. The horizontal lines indicate opening and closing of the shutter to the Pt target. The colour bars indicate the intensity in *Q* space and *G*(*r*) in real space.

Before the PDFs can be obtained from the coherent sample scattering, *I*_c_(*Q*), the 1D azimuthally integrated intensities must be normalized and corrected for incoherent scattering and background contributions. The integrated intensities and resulting PDFs for a sample sputtered at 5 × 10^−2^ mbar, 12 W and substrate temperature of 300 K are shown in Fig. 3 ▸. In the diffraction pattern in Fig. 3 ▸(*a*) the broad peak below 2 Å^−1^ stems from the fused quartz background. It decreases in intensity with increasing film thickness because of the finite penetration depth of the beam in grazing incidence. Hence, each diffraction pattern needs to be assigned a varying background scale. Ideally, a model-free approach should be applied to extract the decreasing level of the background intensity or any other layers that might be present. Multivariate curve resolution by alternate least-squares (MCR–ALS) and principal component analysis have both recently been shown to be effective in extracting phase information in both real space and reciprocal space in complex systems (Staniuk *et al.*, 2014 ▸, 2015 ▸; Chapman *et al.*, 2015 ▸; Birkbak *et al.*, 2017 ▸). Here, MCR–ALS was applied to the GIRXD patterns, assuming non-negativity in intensities and component concentrations, to recover the varying scale of the otherwise fixed substrate signal. Two components were used for the MCR–ALS analysis (for more details, see the Supporting information). The resulting profile of the background level was applied to the averaged background scattering signal obtained before deposition was started. In this way PDFs free of any background were obtained as shown as a function of real space and time in Fig. 3 ▸(*b*). As expected, the signals of the polycrystalline Pt-film increase in intensity with time in both reciprocal and real space. No major changes in the short-range order were observed. As can be seen from the medium-range region, the crystallite sizes clearly increase, as will be discussed below. Since the sputter rate was ∼1.1 Å s^−1^ assuming a constant sputter rate during deposition, a high-quality PDF was obtained after 30 s of deposition, well before the film had reached a thickness of 3 nm.

### 4.3. Modelling the PDFs   

**Figure 4:**
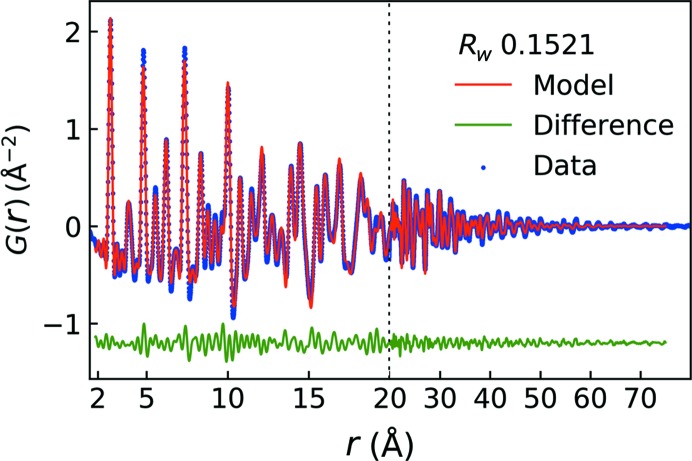
Fit of a 25.4 nm Pt thin film deposited *in situ* with an exposure time of 0.5 s.

To determine the instrumental resolution an identical blank substrate was partly covered with a CeO_2_ powder standard, in order to replicate the measuring conditions as closely as possible (Dippel *et al.,* 2019 ▸). The high-energy X-rays and grazing-incidence geometry effectively caused the sample footprint to be of the order of 5 mm, comparable in dimensions with the total sample–substrate length (10 mm). The *Q* resolution dampening and broadening factors, *Q*_damp_ and *Q*_broad_, respectively, were found to be 0.030 and 0.045, respectively. The PDFs were fitted in *PDFgui* (Farrow *et al.*, 2007 ▸) in the range between 1.75 and 75 Å. The refined parameters were scale factor, lattice parameter, correlated motion (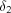), spherical particle diameter and 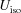. The modelled PDF of the last collected frame is shown in Fig. 4 ▸, corresponding to a film thickness of 25.4 nm. It is noted that a remarkably good fit is obtained (*R_w_* = 15.2%) when taking into account the film thickness. The refinement gives the following values: *a* = 3.928 (2) Å, *U*_iso_ = 0.0071 (7) Å^2^, 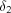 = 5 (1) Å and spherical particle diameter = 10.6 (3) nm.

**Figure 5:**
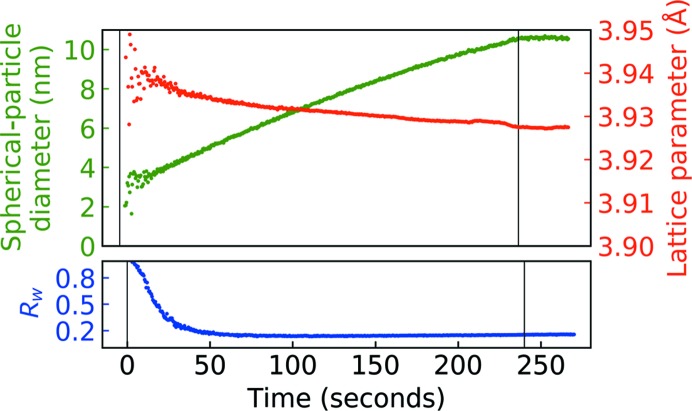
Particle diameter, lattice parameter and *R_w_* from sequentially refining the PDFs of a Pt film deposited at ∼1.1 Å s^−1^ (see main text). The PDFs were refined from the last frame and backwards. Vertical lines indicate opening and closing of the shutter to the Pt target.

The time-resolved PDFs were fitted by a sequential fitting procedure starting from the last PDF and backwards. The fitting procedure was stable throughout the whole dataset, although for the data from the first 20 s of deposition the fitted parameters are highly scattered, see Fig. 5 ▸. This is also reflected in the *R_w_* starting off at ∼0.8 in the early stages but quickly stabilizing at or below *R_w_* = 0.20 when ∼3–4 nm of film were deposited. The lattice parameter steadily decreased during deposition and approached the unstressed Pt lattice constant of 3.9231 Å (Lide, 2005 ▸). The spherical particle diameter grew with near-linear behaviour within the time period of the present experiment. The size stabilized at 10.6 (3) nm when deposition was interrupted at 240 s, suggesting that the particle coarsening is facilitated by the impact energy of the sputtered particles.

**Figure 6:**
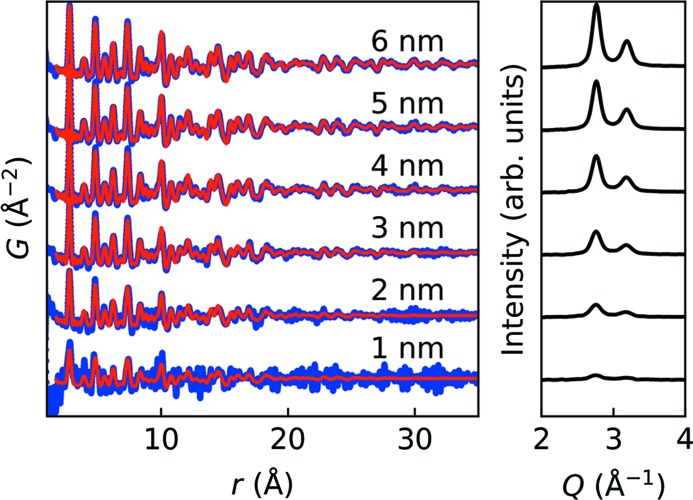
Observed PDFs (blue), modelled PDFs (red) and GIXRD (right plot) in 1 nm intervals up to 6 nm. *R_w_* values in order of increasing thickness were 0.845, 0.489, 0.254, 0.206, 0.183 and 0.161, showing that above ∼3 nm the PDFs were well behaved.

In Fig. 6 ▸ the modelled PDFs are compared with the observed PDFs in intervals of 1 nm up to an estimated thickness of 6 nm (as extrapolated from XRR measured subsequent to the measurement). Noise and termination errors originating from the Fourier analysis from *Q* space to real space are clearly unfavourable to the PDF especially for the first two datasets. Nevertheless, the Pt–Pt correlations are clearly observed in the PDF of the 2 nm thin film. The fit quality is mirrored in the behaviour and stabilization of the refined parameters after about 25 s, which corresponds to an estimated film thickness of 3 nm.

## 5. Conclusions   

We have developed a novel sputter deposition unit, which allows PDF measurements on thin films to be taken during RF magnetron sputter deposition. The PDFs were collected with ∼100 keV photon-energy X-rays in grazing incidence. The substrate scattering background was determined by the model-independent MCR–ALS method. Subsequently, the as-scaled background signal was subtracted from the sample scattering intensity before transformation to PDF. Polycrystalline Pt films without texture were deposited up to a thickness of ∼25 nm. The time-resolved PDFs were modelled to reveal a grain coarsening during the entire deposition process. Furthermore, the lattice parameter had a slight tensile strength that approached bulk Pt with increasing film thickness. It was possible to obtain reliable and clear PDFs of Pt thin films down to a thickness of about 3 nm which is unprecedented. In this way, it is now possible to measure and quantify PDFs during the early stages of thin-film deposition *in situ* under real conditions to gain understanding of transient properties or local- and medium-range order in thin films.

## Supplementary Material

Supporting information. DOI: 10.1107/S2052252519001192/fc5030sup1.pdf
