# Supplementary material for: Time-resolved grazing-incidence pair distribution functions during deposition by radio-frequency magnetron sputtering
Source: IUCrJ. 2019 Feb 23;6(Pt 2):299–304. doi: 10.1107/S2052252519001192 (PMC6400190; doi:10.1107/S2052252519001192)
Supplement: Supplementary file 1 [file m-06-00299-sup1.pdf]

IUCrJ

Volume 6 (2019)

Supporting information for article:

Time-resolved grazing-incidence pair distribution functions during deposition by radio-frequency magnetron sputtering

Martin Roelsgaard, Ann-Christin Dippel, Kasper Andersen Borup, Ida Gjerlevsen Nielsen, Nils Lau Nyborg Broge, Jan Torben Röh, Olof Gutowski and Bo Brummerstedt Iversen

S1. Instrumentation

A side view and rendered CAD drawings from the front and side are shown in fig. S1. When the vacuum chamber is used offline a glass flange is installed in place of the Kapton window.

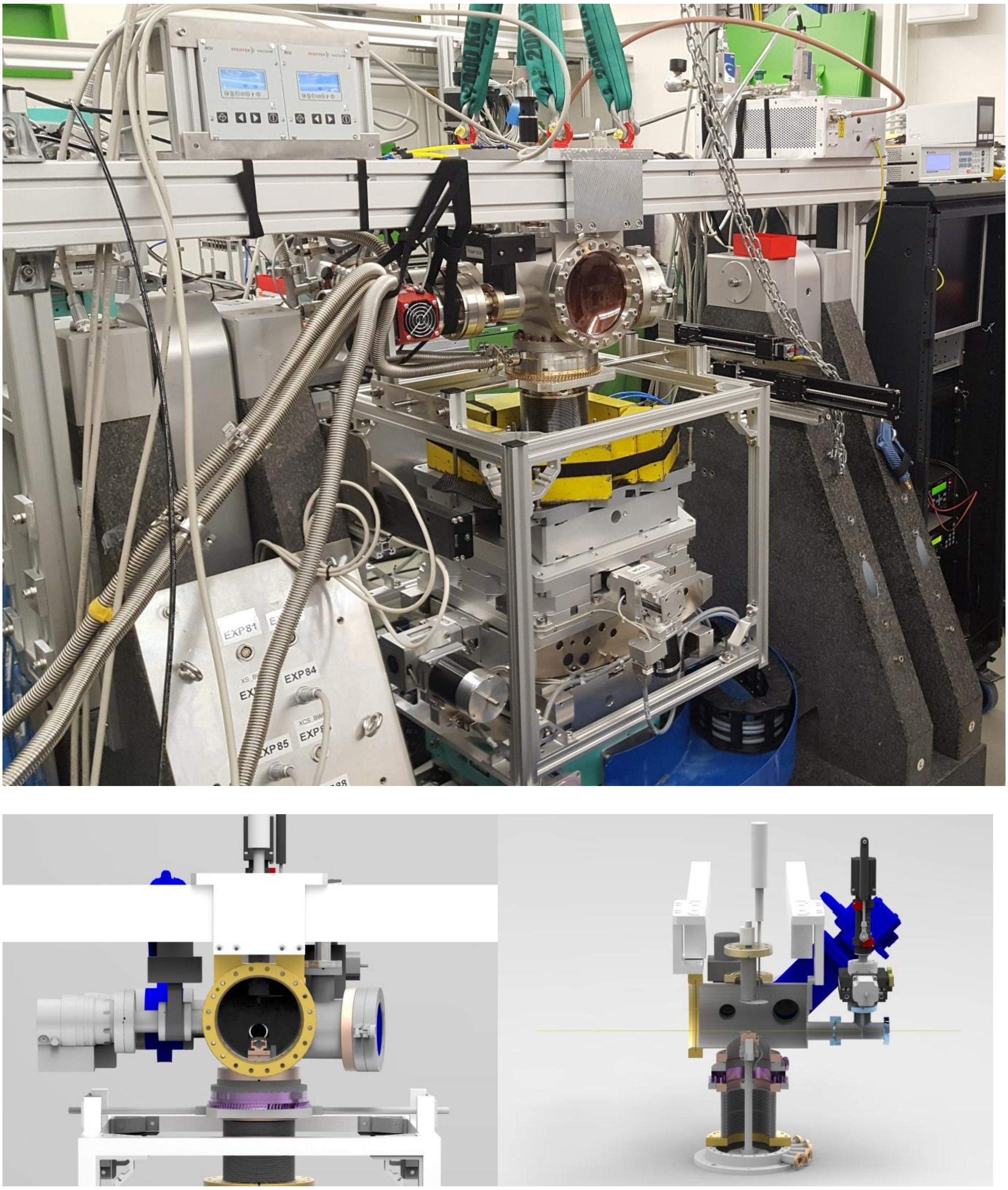

Figure S1 Picture of the vacuum chamber (top) and CAD renderings from front and side.

S2. Pole figures

In figure S2 the pole figure of Pt thin film with fiber texture is shown. The measurement was performed on a Rigaku SmartLab system with Co Kα source and parallel beam option with 0.5x0.1 mm^2 beam size. Pole figures are measured at a fixed 2θ while varying tilt and in-plane rotation. A ring-like pattern is characteristic of a fiber texture with a fixed orientation of the plane in one direction but random orientation in-plane.

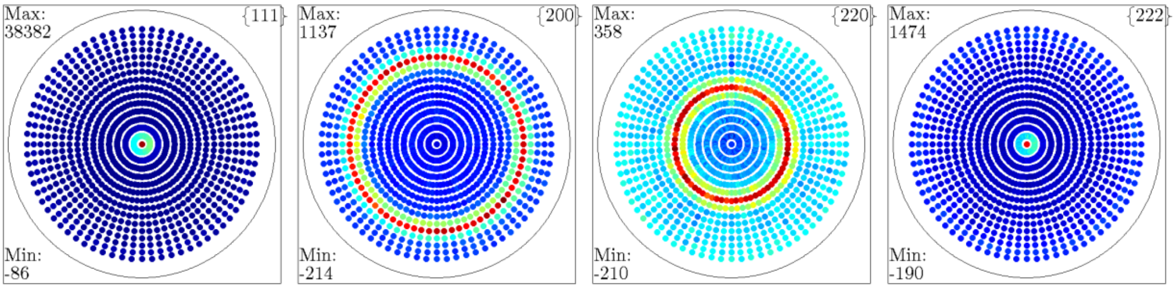

Figure S2 Pole figures for the (111), (200), (220) and (222) reflections of Pt.

S3. Background subtraction

The signal/background intensity is correlated to the thickness of the film, requiring the correct ratio between background and film must be identified for each frame to obtain *S(Q)* and *f(Q)* before Fourier analysis. MCR-ALS was performed with two components assuming non-negativity. The components and their “scale factor” are shown below (solid lines). The MCR-ALS component concentrations were re-scaled to correspond to the average background (between 1 and 0.34) and shown compared to the ratio between substrate structure factor and Pt (111) reflection intensities.

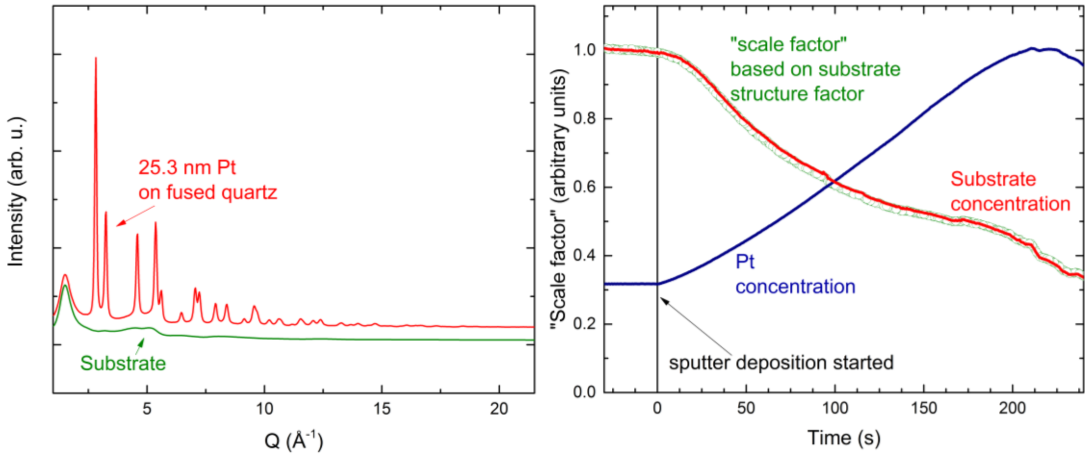

Figure S3 (a) Substrate signal averaged from frames prior to film deposition. (b) comparison of (111)-reflection to the substrate peak for each point in time during the experiment (green circles) and scaled component concentration as extracted by MCR-ALS.
